# Impact of in vitro exposure to 5G-modulated 3.5 GHz fields on oxidative stress and DNA repair in skin cells

**DOI:** 10.1038/s41598-025-15090-w

**Published:** 2025-08-25

**Authors:** Jana Haidar, Patricia Nabos, Rosa Orlacchio, Annabelle Hurtier, Florence Poulletier de Gannes, Jérome Rambert, Muriel Cario-André, Francois Moisan, Hamid-Reza Rezvani, Isabelle Lagroye, Philippe Leveque, Delia Arnaud-Cormos, Yann Percherancier

**Affiliations:** 1 Bordeaux University, CNRS, IMS Laboratory, UMR5218, 33400 Talence, France; 2 EPHE, Paris Sciences et Lettres Research University, 75006 Paris, France; 3 Bordeaux University, Inserm, BRIC, UMR 1312, 33076 Bordeaux, France; 4 Limoges University, CNRS, XLIM, UMR 7252, 87000 Limoges, France; 5 Institut Universitaire de France (IUF), Paris, France

**Keywords:** Radiofrequency electromagnetic fields (RF-EMF), Fifth generation (5G), Bioluminescence resonance energy transfer (BRET), Cellular stress response, Reactive oxygen species (ROS), Oxidative stress, DNA damage, Cyclobutane pyrimidine dimer (CPD), Nucleotide excision repair (NER), Environmental impact, Cell biology

## Abstract

The rapid deployment of fifth-generation (5G) wireless networks has raised societal concerns regarding potential biological effects, particularly on human skin, due to the use of higher carrier frequencies that penetrate tissue less deeply. Consequently, whether 5G-modulated radiofrequency (RF) electromagnetic fields (EMFs) at 3.5 GHz affect oxidative stress and DNA repair in skin cells remains an open question. Using genetically encoded Bioluminescence Resonance Energy Transfer (BRET)-based biosensors targeted to the cytoplasm and mitochondria, we assessed whether exposure of human fibroblasts to 5G RF-EMF at specific absorption rates (SAR) of 0.08 and 4 W/kg for 24 h could alter basal reactive oxygen species (ROS) levels or potentiate the effects of known ROS inducers, including H₂O₂, Kp372-1, and Antimycin A. We also evaluated whether pre-exposure to 5G RF-EMF could induce an adaptive response (AR), by modulating ROS production following a subsequent challenge with arsenic trioxide (As₂O₃). Additionally, we investigated the impact of combined RF-EMF and ultraviolet-B (UV-B) exposure on the formation and repair of cyclobutane pyrimidine dimer (CPD) lesions in HaCaT keratinocytes. Our results showed no significant effect of 5G RF-EMF exposure, either alone or in combination with chemical ROS inducers, on oxidative stress markers in either compartment. Likewise, RF-EMF exposure did not induce an adaptive response to oxidative challenge, nor did it alter the kinetics or the efficiency of CPD repair by the nucleotide excision repair (NER) pathway. These findings support the conclusion that the exposure to 5G RF-EMF at 3.5 GHz up to 4 W/kg does not induce oxidative stress or impair DNA repair efficiency in human skin cells, within the experimental conditions tested.

## Introduction

Over the past two decades, extensive research has been conducted to explore the potential health effects of exposure to radiofrequency (RF) electromagnetic fields (EMFs), ranging from 100 kHz to 300 GHz, particularly due to the rapid expansion of wireless communication systems. During this time, the mechanism by which RF waves interact with living matter has been thoroughly characterized. Unlike high-energy photons, which can directly induce chemical reactions and DNA breaks, RF-EMF photons lack sufficient energy to cause such effects. Instead, RF waves primarily cause heating in biological tissues through dielectric relaxation, a process where the oscillation of polar molecules in response to the electromagnetic field generates heat^1^.

To protect against this thermal effect, standards have been established by the International Commission on Non-Ionizing Radiation Protection (ICNIRP), defining exposure limits often expressed as the specific absorption rate (SAR) in Watts per kilogram (W/kg) for frequencies below 6 GHz. Nonetheless, societal concerns about potential « non-thermal » effects have arisen, and the International Agency for Research on Cancer (IARC) classified RF-EMF as Group 2B carcinogens in May 2011, meaning they are possibly carcinogenic to humans^2^. The only remaining question is whether RF-EMF exposure can trigger potential non-thermal effects. This question continues to drive research, especially as new generations of telecommunication technologies emerge.

The fifth generation (5G) of wireless network has emerged with the purpose of improving the 4G LTE technology by resolving issues linked to reliability and latency^3,4^. To resolve these problems, it was required adding new frequency bands alongside the frequencies used for 2G, 3G, and 4G. The new frequency bands are 3.5 GHz and 26 GHz. The former, also known as C-band, is critical component of the present 5G since it balances vast internet coverage with rapid speeds. On the other hand, the 26 GHz band has a limited ability to penetrate buildings and efficiently propagate signals, making it more suitable for covering specific areas with high data traffic.

The potential genotoxic effects of environmental RF-EMF have been a subject of scientific inquiry for decades. With the rapid expansion of wireless communication technologies, including 5G, concerns regarding the impact of RF-EMF on DNA integrity and cellular health have intensified. Numerous studies have explored whether exposure to these fields can influence the balance of reactive oxygen species (ROS), antioxidant defenses, and DNA repair mechanisms.

Experimental studies have reported that exposure to RF-EMF can induce oxidative stress responses across diverse biological systems. De Iuliis et al.^5^ exposed purified human spermatozoa to 1.8 GHz RF-EMF (SAR 0.4–27.5 W/kg) and observed a dose-dependent increase in mitochondrial ROS and DNA fragmentation. In that study, dosimetry and temperature control were rigorously implemented. Kesari and Behari^6^ studied mobile phone signals (900 MHz, SAR ~ 0.9 W/kg, 2 h/day, 35 days) and found increased testicular oxidative stress and apoptosis in male rats. However, temperature control was only ambient, and animals were housed in a climate-controlled room (24–26 °C). Oksay et al.^7^ reported that Wi-Fi exposure at 2.45 GHz for 30 days induced oxidative stress in rat testes, with increased lipid peroxidation and reduced antioxidant levels; while electric field estimates were used to infer SAR (~ 0.143 W/kg), no direct SAR measurements or temperature monitoring were performed. Kazemi et al.^8^ found increased ROS production in monocytes—but not lymphocytes—after exposure to GSM-modulated fields (900 MHz, SAR 2 W/kg), using a system with controlled dosimetry and temperature regulation. More recently, Pooam et al.^9^ exposed HEK293 cells to 1.8 GHz signals (SAR ~ 2 W/kg), both GSM-modulated and continuous-wave, and observed significant ROS increases with temperature tightly controlled, although no dosimetric modeling was provided.

Yakymenko et al.^10^ reviewed over 100 studies and concluded that low-intensity RF-EMF is a significant oxidative stressor affecting mitochondria and NADH oxidase pathways. However, this review did not evaluate or report on quality of the dosimetry, modulation type, or temperature control of the included studies, and therefore lacked consistency in exposure assessment. Similarly, Di Ciaula^11^ discussed gene expression changes associated with inflammation and oxidative stress due to millimeter-wave RF-EMF, but without providing dosimetric validation or analyzing exposure conditions. Kerna et al.^12^ cited various RF-EMF effects, including oxidative DNA damage and potential metabolic and neurological impacts, although they did not systematically examine the exposure setups or the dosimetry of the referenced studies. Schuermann and Mevissen^13^ conducted a systematic review of oxidative stress related to EMF exposure, concluding that a majority of animal and more than half of the cellular studies showed increased oxidative stress. However, they also emphasized that many studies lacked SAR data, used mobile phones as RF sources, or failed to control for thermal effects. Finally, Henschenmacher et al.^14^ proposed a systematic protocol to standardize biomarker analysis and improve dosimetric consistency in future studies. Collectively, these articles illustrate the heterogeneity in outcomes regarding RF-induced oxidative stress. Discrepancies arise from variations in biological models, exposure duration, modulation, SAR levels, and, critically, from inconsistent or missing dosimetric and thermal characterization. Importantly, many review articles that synthesize these findings do not evaluate these methodological parameters, which limits the strength of their conclusions. Therefore, there remains no scientific consensus, and further well-controlled, dosimetrically validated studies are essential to determine whether RF-EMF, including 5G-related frequencies, affect oxidative stress pathways in biological systems. This position is echoed by recent reports, including the Swedish Scientific Council on Electromagnetic Fields (SSM), which reaffirm that observed oxidative effects in some studies are likely attributed to experimental artifacts or inadequate exposure characterization^15,16^. Importantly, SSM highlights the absence of a biologically plausible mechanism by which low-level, non-thermal RF-EMF exposure could initiate or sustain oxidative stress.

Interestingly, rather than inducing purely adverse effects, some studies have suggested that RF-EMF may trigger an adaptive response (AR) in cells, a fundamental defense mechanism that minimizes genotoxic damage and cell injury by increasing cellular resistance to stress. This phenomenon, observed across prokaryotes, yeast, plants, and mammals, is triggered by exposure to low, non-toxic doses of a given adverse chemical agents, referred to as adaptive doses (AD), which enhance resilience to subsequent higher, toxic doses, known as challenging doses (CD)^17–20^. Such responses allow organisms to cope with various chemicals or physical agents, both natural and anthropogenic (e.g., UV light, ionizing radiation, EMF-RF). Historically, AR has first been described using ionizing radiation. For instance, Fan et al.^21^ used 1 cGy of X rays as AD in human blood peripheral lymphocytes and showed a significantly reduced incidence of 1 Gy X-rays induced chromatid breaks. Concerning bioelectromagnetics studies, Sannino et al.^22^ initially reported that 900 MHz RF-EMF could induce an AR in human peripheral blood lymphocytes exposed to RF-EMF at a SAR of 10 W/kg for 20 h and, 18 h later, challenged with mitomycin C (100 ng/mL) for 3 h. The authors observed a significant decrease in the incidence of micronuclei in cytokinesis-blocked binucleated cells. More recently, Ji et al.^23^ reported that RF-EMF exposure could induce an AR in mouse bone marrow stromal cells, leading to increased DNA repair efficiency after gamma radiation-induced strand breaks. Similarly, Zong et al.^24^ found that pre-exposure to 900 MHz RF fields resulted in reduced DNA damage when cells were later exposed to genotoxic agents like bleomycin. Finally, Falone et al.^17^ observed that RF-EMF induced AR could reduce oxidative DNA damage in neuroblastoma cells when challenged with additional oxidative stressors. These findings suggest that RF-EMF may not necessarily be harmful in all cases but might instead activate cellular defense mechanisms, mitigating DNA damage under specific exposure conditions^25^.

Despite these accumulated observations, a fundamental limitation persists: no molecular mechanism has been definitively identified to explain how RF-EMF at environmental levels could physically interact with biological molecules to induce oxidative stress, ROS formation, genotoxicity or even AR^26,27^. While several authors suggest potential biochemical pathways^10,28^, these remain largely speculative and unconfirmed through direct mechanistic studies^1,26^. The absence of a well-defined physical or biochemical mechanism has relegated research in this field to an empirical approach, where studies rely on detecting oxidative or genotoxic markers without a clear causal framework. This challenge is particularly characteristic of environmental bioelectromagnetics, where many observed effects remain phenomenological rather than being mechanistically elucidated.

In a former study, we assessed the effects of 5G-modulated 3.5 GHz RF-EMF on mitochondrial stress in human fibroblasts and keratinocytes exposed for 24 h at SAR levels of 0.25, 1, and 4 W/kg^29^. The study focused on cell viability, mitochondrial ROS production, and mitochondrial membrane polarization, both under RF-EMF alone and in combination with ultraviolet-B (UV-B) irradiation. A slight reduction in mitochondrial ROS levels was observed in fibroblasts, but not in keratinocytes, following RF exposure at 1 W/kg. By constrast, the combined exposure of RF-EMF and UV-B significantly increased UV-induced ROS production in keratinocytes at SAR levels of 0.25 and 1 W/kg but not at 4 W/kg. However, exposure to 5G RF at 3.5 GHz RF did not impact mitochondrial membrane potential or cell viability in either cell type , suggesting that the observed effects had minor biologically impact at the whole cell level. Here we propose novel approaches utilizing Bioluminescence Resonance Energy Transfer (BRET)-based genetically engineered ROS sensors and biochemical assessment of DNA repair to re-assess the impact of 5G-modulated 3.5 GHz RF signals on oxidative stress, AR and DNA repair in skin cells exposed to SAR levels of 0.08 and 4 W/kg for 24 h.

## Materials and methods

### Plasmids and reagents

**Fig. 1:**
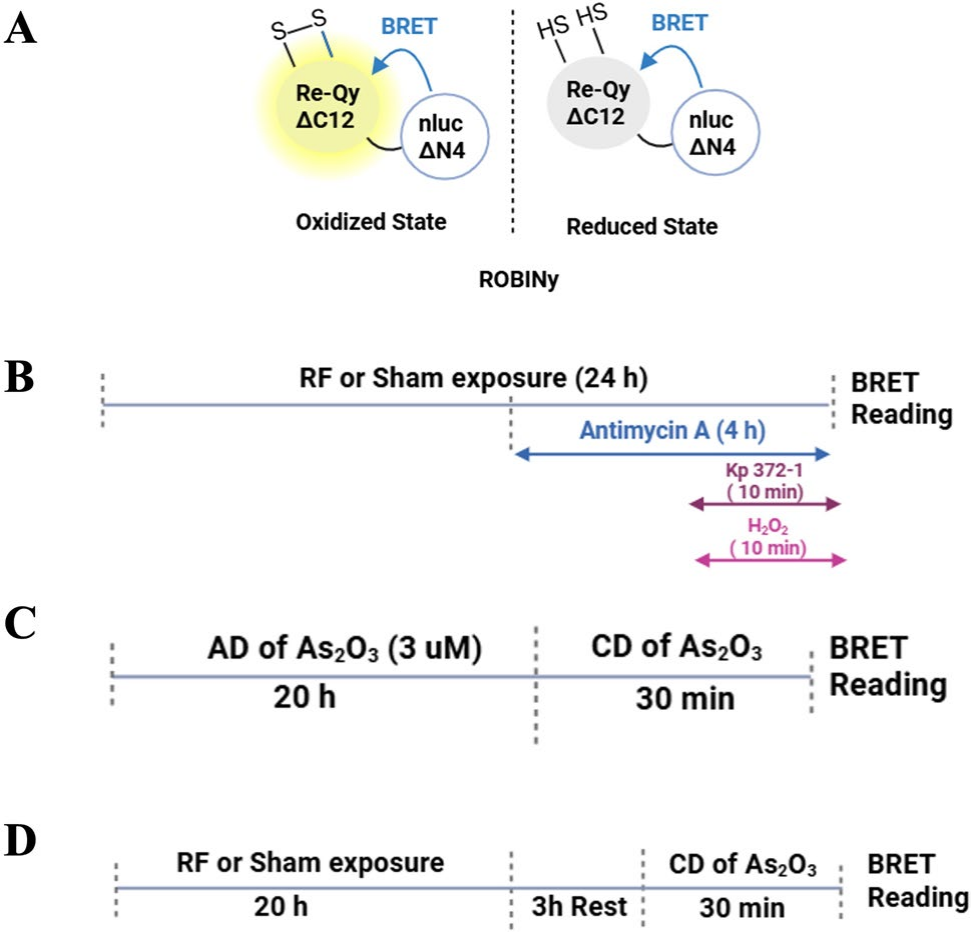
(**A**) Schematic representation of the BRET-based redox-sensitive biosensor ROBINy in both oxidized and reduced states (adapted from Fu et al.^30^). ROBINy consists of nanoLuc ΔN4 (nluc lacking the N-terminal 4 amino acids) and Re-Qy ΔC12 (a redox-sensitive fluorescent acceptor lacking the C-terminal 12 amino acids). Under oxidative conditions, disulfide bonds form within Re-Qy, increasing its fluorescence and thus enhancing the energy transfer from nluc to Re-Qy, resulting in a higher BRET ratio. (**B**–**D**) Timeline of experiments conducted in XP6BE human fibroblasts transfected with cytoplasmic or mitochondrial ROBINy. B) Direct ROS response assay: cells were sham- or RF-EMF-exposed (5G-modulated, 3.5 GHz) for 24 h. During the last 10 min of exposure, cells were treated with either H₂O₂ (0–100 mM) or Kp372-1 (0–100 µM). Alternatively, cells were treated with Antimycin A (0–300 µM) during the final 4 h of the exposure period. (**C**) Positive control for adaptive response: cells were pretreated with arsenic trioxide (As₂O₃, 3 µM, 20 h; adaptive dose, AD), then challenged with increasing concentrations (challenging dose, CD, 30 min). (**D**) RF-induced adaptive response assay: cells were exposed to RF-EMF or sham-exposed for 20 h, rested for 3 h, then challenged with CD of As₂O₃ for 30 min. BRET was measured immediately after the end of the RF exposure and/or chemical treatment.

To assess ROS production in skin cells, we outsourced the cloning of the ROBINy sensor^30^ (Fig. 1A), into the pcDNA3.1(+) mammalian expression vector (Genscript). The following chemicals were used: Akt Inhibitor KP372-1 (Medchem, HY-15673), hydrogen peroxide (H_2_O_2_) (Sigma, 216763, 30 wt. %in H_2_O, ACS reagent), Antimycin A (from Streptomyces sp; Sigma A8674), Arsenic trioxide (As₂O₃ , A1010), and Furimazine (Nanolight Technology (Pinetop, AZ, USA)).

### RF-EMF setup and cell exposure conditions

Cells were exposed to 5G-modulated RF-EMF at 3.5 GHz using a system specifically designed to ensure precise and reproducible in vitro RF exposure. This system, characterized through combined experimental measurements and numerical simulations^31^, consisted of three independent 150-L stainless-steel incubators (BINDER GmbH, Tullingen, Germany), each operating as a reverberation chamber that maintained physiological conditions (37 °C, 5% CO₂) throughout the exposure period. These metallic structures provided a statistically homogeneous, isotropic, and randomly polarized EMF distribution generated via mechanical stirring of the field. Exposure conditions were assigned randomly across the three chambers following a blind protocol.

RF signals at 3.5 GHz were emitted via a printed antenna installed within each chamber. To achieve mechanical stirring of the electromagnetic field, a metallic stirrer consisting of 5 rectangular blades was attached to a rod fixed on a motor placed at the top of each chamber. Each stirrer was rotated by a motorized stage (PRM1/MZ8; Thorlabs Inc.), controlled via a K-Cube DC servo controller (KDC101; Thorlabs), ensuring rotation throughout the exposure and located outside the incubator. The signal generation unit located externally to the incubator comprised the following components: signal generator (SMBV100A, Rohde & Schwarz, Munich, Germany) with signal type 5G NR (release 15, Digital Standards SMBVB‐K444; Rohde & Schwarz) with FDD duplexing, QPSK modulation and 100 MHz channel bandwidth; high-gain amplifier (ZHL-16W-43+ , Mini-Circuits, NY, USA); power circulator (PE83CR1005, Pasternack, CA, USA); bidirectional coupler (ZGBDC30-372HP+ , Mini-Circuits, NY, USA). To obtain the desired exposure levels (sham, 0.08, and 4 W/kg), two directional couplers (C1-C74-06N, C1-C74-10N, RDmicrowaves, NJ, USA and a BW-N1W5+ attenuator, Mini-Circuits, NY, USA) were inserted in the setup. Power was split into the three chambers using three dual RF SP4T switch matrix (RC-2SP4T-26, Mini-Circuits, NY, USA). Incident and reflected power were continuously monitored via an Agilent N1912A power meter connected to the coupler.

A custom-designed plastic holder with five shelves was used to accommodate both 6-well and 96-well tissue culture plates in each chamber. To guarantee the correct dosimetry, the chamber was loaded with a total of 10 plates including four 6-well and six 96-well plates filled with 2 mL and 200 μL of cell culture medium per well, respectively^31^. To maintain appropriate humidity during incubation, a water reservoir was placed at the bottom of each chamber.

Local SAR was experimentally retrieved through temperature measurements of the RF-EMF induced heating recorded with a fiber-optic probe (Luxtron One, Lumasense Technologies, CA, USA). For exposures at a SAR of 4 W/kg, temperature compensation was implemented to counteract RF-EMF induced heating by lowering the incubator setpoint by 2 °C (for 96-well plates) or 1 °C (for 6-well plates) to maintain constant physiological temperature at the sample level during RF-EMF exposure. The steady maintenance of 37 °C was monitored throughout the experiments using the Luxtron fiber-optic probe. Cells were exposed to 5G-modulated 3.5 GHz RF-EMF for 24 h to asses ROS production (Fig. 1B), for 20 h to evaluate AR (Fig. 1C and D), and up to 48 h to monitor DNA damage repair (Fig. 6A). Exposures were carried out in 96-well tissue culture plates (ROS experiments) or 6-well plates (DNA Damage experiments), at SAR levels of 0.08 or 4 W/kg. Sham exposures were performed in parallel under identical conditions, except that the RF-EMF signal generator remained off.

### Cell culture

For ROS monitoring experiments, the SV40-immortalized skin fibroblast cell line XP6BE^32^, derived from a 19-year-old female patient with xeroderma pigmentosum (complementation group D), was obtained from the Coriell Institute (Camden, NJ, USA). Cells were cultured in Dulbecco’s Modified Eagle Medium/Nutrient Mixture F-12 with high glucose (DMEM-F12 ; Sigma-Aldrich, D6429) supplemented with 10% fetal bovine serum (FBS), 100 units mL^-1^ penicillin and streptomycin, and 1.43 mg/mL glucose (Gibco™, glucose solution, Ref.A24940-01).

For DNA damage experiments, HaCaT cells, spontaneously transformed human keratinocytes^33^,were purchased from Cell Line Service GmbH (Eppelheim, Germany) and cultured in high-glucose DMEM (Sigma-Aldrich, D6429) supplemented with 10% FBS, 100 units mL^-1^ of penicillin and strepromycin, and 1.78 mg/mL glucose. The cells were seeded in 6-well plates at a density of 200,000 cells per well (100,000 cells/mL in a total volume of 2 mL) and incubated overnight.

Cells were maintained at 37 °C in a humidified atmosphere containing 5% CO_2_.

### Transfections

Transient transfections were performed in T75 flasks using linear polyethyleneimine (PEI, MW 25,000; Polysciences, Inc., 23,966) at a DNA:PEI ratio of 1:5^34^. A total of 15 μg of DNA was used per flask, consisting of 2.25 μg of the ROBINy ROS sensor (Fig. 1A) construct and 12.75 μg of pcDNA3.1( +) empty vector. Following overnight incubation, cells were detached, resuspended in phenol red-free DMEM (Thermo Fisher Scientific, 21063-029), and seeded into white 96-well plates with clear bottoms (Greiner Bio-One, Courtaboeuf, France) at a density of 40,000 cells/well (200 μL/well of a 2 × 10^5^ cells/mL suspension). Cells were incubated for 24 h at 37 °C, with or without RF-EMF exposure, prior to BRET measurement using a Tristar2 luminometer (Berthold Technologies, Bad Wildbad, Germany).

### UV-B irradiation

HaCaT keratinocyte cells were irradiated with UV-B light at a dose of 20 mJ/cm^2^ using a Bio-Link® BLX-E312 system (Vilber Lourmat; Fisher Bioblock), which emits at a peak wavelength of 312 nm. To establish the optimal irradiation dose, cell viability was assessed using the trypan blue exclusion assay following exposure to increasing doses of UV-B. To minimize the formation of photoactive byproducts, the culture medium was replaced with Hanks’ Balanced Salt Solution (HBSS; 137 mM NaCl, 5.4 mM KCl, 1.26 mM CaCl₂, 0.81 mM MgSO₄, 4.2 mM NaHCO₃, and 5.6 mM glucose) during irradiation. Immediately after UV-B exposure, the HBSS was replaced with fresh growth medium, and the cells were returned to standard culture conditions. The UV-B irradiation was followed by exposure or sham exposure (incubated in the absence of RF-EMF) to 5G-modulated 3.5 GHz RF-EMF at SAR levels of 0.08 and 4 W/kg for up to 48 h. Samples were collected at 3, 6, 24, and 48 h post-UV exposure, snap-frozen, and stored until further analysis.

### Chemical treatment and BRET measurements

To assess whether prior RF exposure could impact ROS production in XP6BE cells, cells were either sham-exposed or exposed to 5G-modulated 3.5 GHz RF-EMF signal at SAR level of 0.08 or 4 W/kg for 24 h. During the final 10 min or 4 h of either sham or RF-exposure, cells were treated with increasing concentrations of H₂O₂ (0–100 mM, 10 min), Kp372-1 (0–100 µM, 10 min), or Antimycin A (0–300 µM, 4 h), as depicted in Fig. 1B.

For the AR assays performed in XP6BE cells, cells were pretreated for 20 h with an AD of As₂O₃, followed by direct treatment with increasing CD of As₂O₃ (Fig. 1C). Alternatively, cells were either sham-exposed or exposed to 5G-modulated 3.5 GHz RF-EMF at SAR levels of 0.08 or 4 W/kg for 20 h, followed by a 3-h rest period, and then treated with increasing CD of As₂O₃ (Fig. 1D).

BRET reading was initiated by replacing culture medium of each well with HBSS and injecting 5 μM furimazine (NanoGlo®, Promega). BRET signals were then acquired immediately using a TriStar2 LB942 plate reader (Berthold Technologies, Bad Wildbad, Germany) maintained at 37 °C. Emission intensities were recorded using filter sets centered at 460 ± 20 nm (nanoluciferase) and 540 ± 20 nm (Re-Qy, the chromophore part of ROBINy^30^).

BRET signals were calculated using the following formula:

1 $$BRET=\frac{{I}_{Re-Qy }}{{I}_{nLuc}}$$

where *I*_*nLuc*_ and *I*_*Re-Qy*_ represent the luminescence intensities measured for the donor nanoluciferase (nLuc) and the acceptor (Re-Qy), respectively. Due to partial spectral overlap, a fraction of the donor signal may bleed into the acceptor detection window. To account for this, net BRET values were computed by subtracting the BRET ratio of cells transfected with donor-only constructs:

2 $$\text{BRET net}=BRET brut-BRET nLuc$$

### Data processing

Dose–response data were analyzed using GraphPad Prism v10.4.2 (GraphPad Software, La Jolla, CA, USA). Sigmoidal concentration–response curves were fitted using the four-parameter logistic equation:

$$Y=Bottom +\frac{Top-Bottom}{1+{10}^{({Log EC}_{50}-X)}}$$

where X is the logarithm of the agonist concentration, *Y* is the BRET signal, *Bottom* and *Top* are the minimum and maximum asymptotes, respectively. EC₅₀ values represent the concentration required to reach 50% of the maximal response. Potency is expressed as pEC₅₀ (− logEC₅₀), and efficacy as the difference between top and bottom. All values are reported as mean ± standard error of the mean (SEM).

### Immuno-dot blot analysis

CPDs were quantified using an immuno-dot blot assay as previously described^35^. Briefly, cell pellets were lysed overnight at 65 °C in Direct PCR Lysis Reagent (Euromedex, Souffelweyersheim, France) supplemented with 2% proteinase K (Sigma-Aldrich). Genomic DNA was then extracted via sodium acetate/ethanol precipitation and quantified using a nanodrop spectrophotometer (Thermo Fisher Scientific, Waltham, MA, USA). A total of 500 ng of genomic DNA was mixed with 1% SYBR Green (Brilliant III Ultra-Fast SYBR; Agilent Technologies, Les Ulis, France) and applied to a Hybond N+ nitrocellulose membrane (Amersham, Little Chalfont, UK) using a dot-blot apparatus (Bio-Rad Laboratories, Cat# 170-6545, 170-6547). Membranes were blocked for 1–2 h at room temperature in Tris-buffered saline (TBS) containing 20 mmol/L Tris, 5% non-fat dry milk, and 0.5% Tween-20 (pH 7.6). After blocking, membranes were incubated overnight at 4 °C with a monoclonal anti-CPD antibody (Cosmo Bio Co., Ltd., Cat# CAC-NM-DND-001; Tokyo, Japan) at a 1:1,000 dilution. Following several washes with TBS, membranes were incubated for 1 h with a horseradish peroxidase (HRP)-conjugated secondary antibody (1:2,000 dilution; Vector Laboratories, Cat# PI-2000; Newark, CA, USA). Blots were developed and imaged using an enhanced chemiluminescence detection system (Bio-Rad, Hercules, CA, USA). SYBR green fluorescence was used as a loading control to verify equal DNA loading across all samples.

### Cellular impedance assay

Cellular impedance measurement of global cellular activity was monitored in a humidified incubator at 5% CO_2_ and 37 °C using the xCELLigence apparatus (Agilent, Santa Clara, United States). Using this setup, changes induced in the local ionic environment at the electrode/solution interface yield an increase in electrode impedance. Changes in cell morphology and/or adhesion that modulate the physical contact between cells and electrodes are reflected in impedance changes. The change in impedance is reported as a dimensionless parameter called Cell Index (CI) according to the equation:

$$CI=\frac{\text{impedance at point n - impedance without cells}}{\text{nominal impedance}}$$

### Statistical analysis

Statistical significant differences between sham- and RF-exposed conditions were analyzed using the nonparametric Kruskal–Wallis test. When positive, the Mann–Whitney test was used to compare each group against the no-field or control group. For comparison between two groups, the Mann–Whitney test was used. Probabilities of *p*-values < 0.05 were considered statistically significant. The number of biological replicates (n) is indicated in the figure legends.

## Results

### Effects of 5G‐modulated 3.5 GHz RF‐EMF exposure and ROS inducers on cytoplasmic and mitochondrial ROS production in XP6BE fibroblasts

To investigate the potential impact of 3.5 GHz RF-EMF on ROS production, we employed the BRET sensor ROBINy, specifically designed to monitor redox dynamics in live cells^30^. This probe consists of a bioluminescent donor (nluc) and a redox-sensitive fluorescent acceptor (Re-Qy), which undergoes significant spectral changes in response to alterations in the cellular redox state. Under oxidizing conditions, the redox-active cysteine residues in the Re-Qy domain form disulfide bonds, altering the chromophore’s absorption spectrum and enhancing the efficiency of the non-radiative energy transfer from the nLuc to Re-Qy. This change is reflected by the increase of Re-Qy emission and the concomitant increase of the ROBINy BRET ratio (Fig. 1A).

**Fig. 2:**
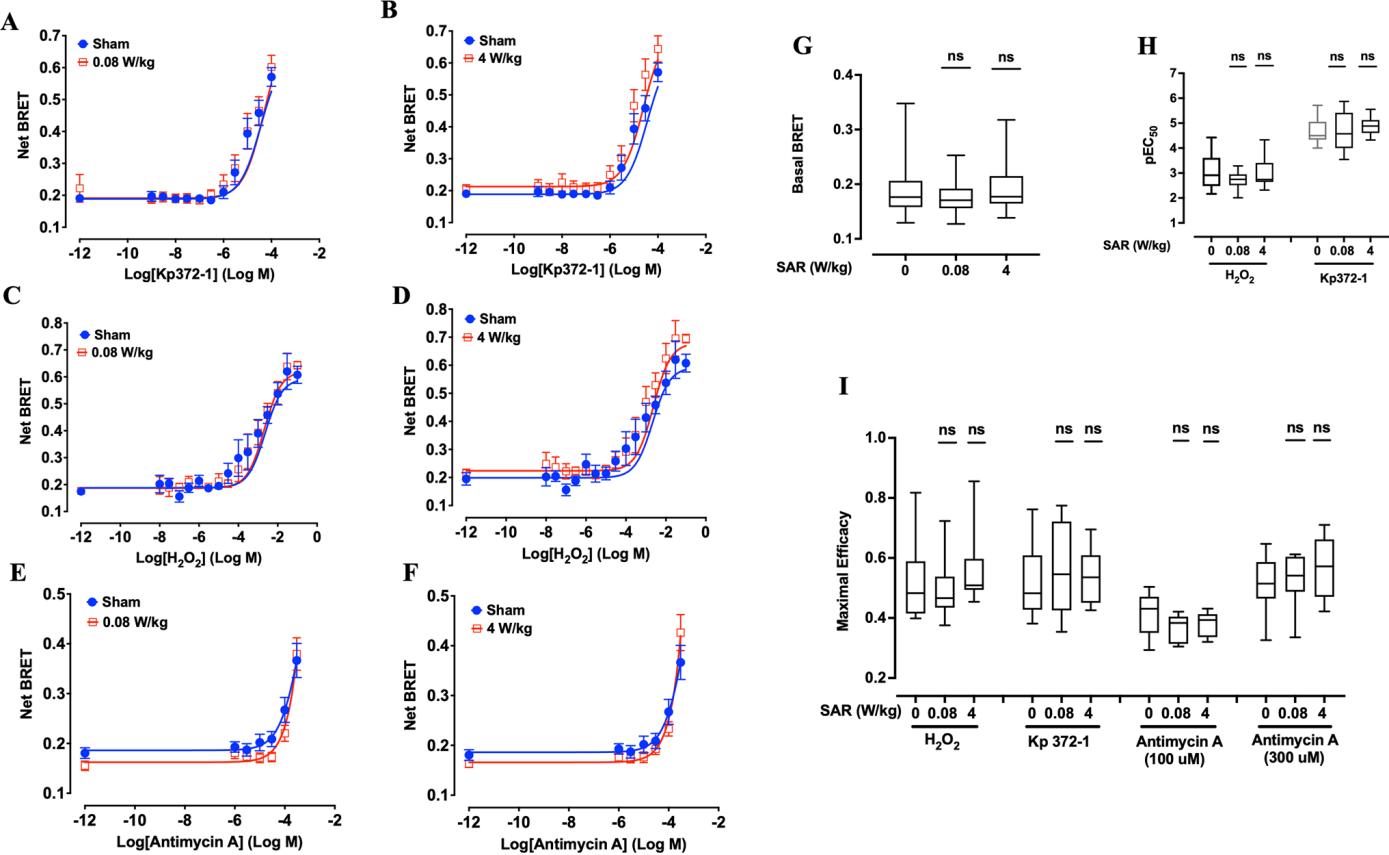
Effect of 24-h exposure to 5G-modulated 3.5 GHz RF-EMF on ROS production induced by chemical stressors in XP6BE human fibroblasts transfected with the cytoplasmic ROBINy BRET probe. Cells were exposed for 24 h to sham or RF-EMF at 0.08 W/kg (**A**, **C**, **E**) or 4 W/kg (**B**, **D**, **F**), and challenged with increasing concentrations of Kp372-1 (**A**, **B**), H₂O₂ (**C**, **D**), or Antimycin A (**E**, **F**). Chemical stressors were applied during the final 10 min (for Kp372-1 and H₂O₂) or final 4 h (for Antimycin A) of RF exposure, as illustrated in Fig. 1B. BRET signals were recorded immediately after RF-EMF exposure, allowing real-time assessment of ROS dynamics. (**G**–**I**) Box plots display quantitative analysis of basal BRET signal (**G**), pEC50 values of H_2_O_2_ and Kp372-1 (**H**), and maximal efficacy for each ROS inducer (**I**) across the different SAR conditions. Due to its limited potency, Antimycin A efficacy was calculated based only on responses to the two highest concentrations (100 and 300 µM). Statistical analysis was performed using Kruskal–Wallis test. No significant differences were observed between RF-exposed and sham groups (n = 8–9 per condition). n.s.: not significant.

**Fig. 3:**
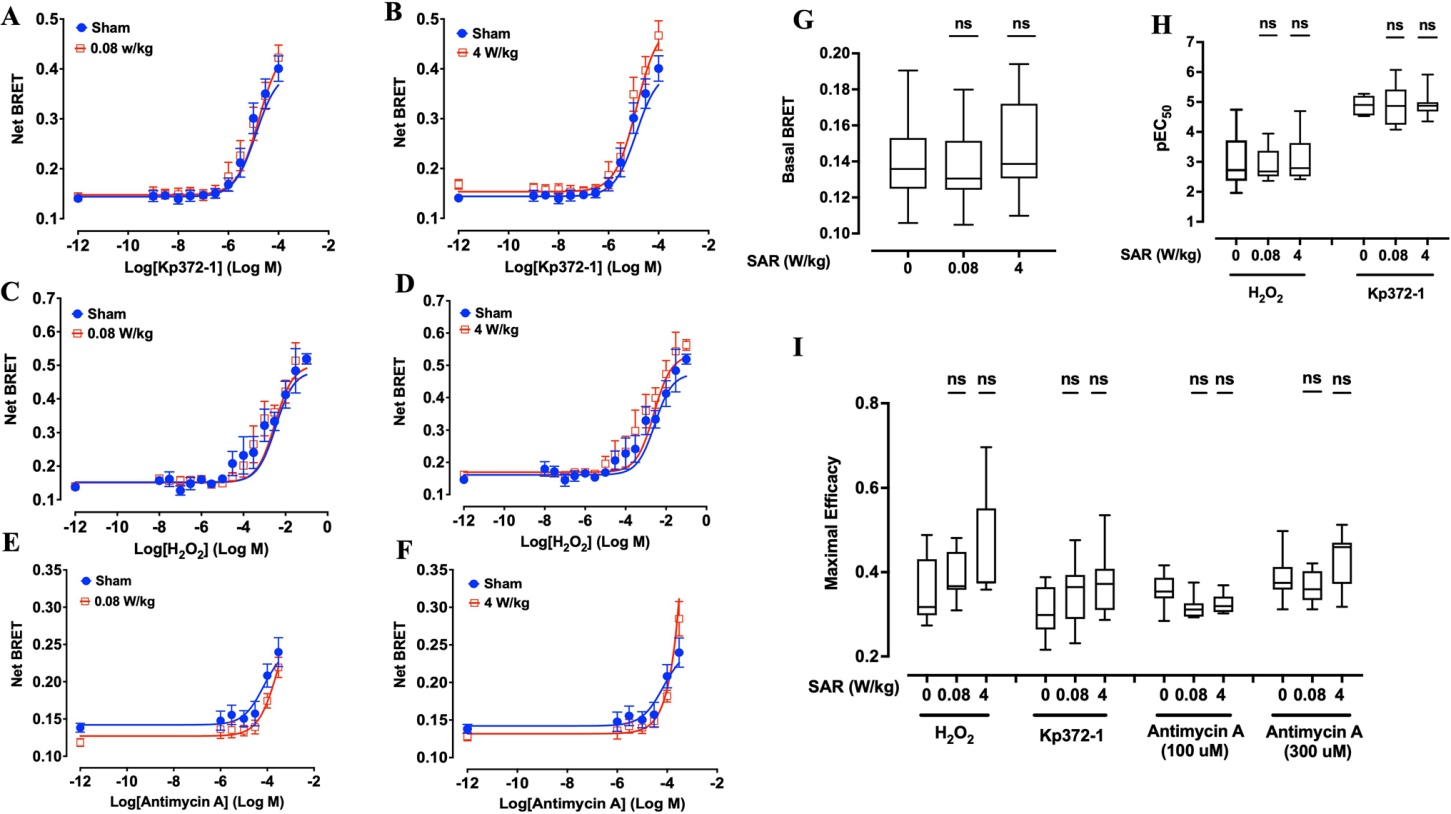
Effect of 24-h exposure to 5G-modulated 3.5 GHz RF-EMF on ROS production induced by chemical stressors in XP6BE human fibroblasts transfected with the mitochondrial ROBINy BRET probe. Cells were exposed for 24 h to sham or RF-EMF at 0.08 W/kg (**A**, **C**, **E**) or 4 W/kg (**B**, **D**, **F**), and challenged with increasing concentrations of Kp372-1 (**A**, **B**), H₂O₂ (**C**, **D**), or Antimycin A (**E**, **F**). Chemical stressors were applied during the final 10 min (for Kp372-1 and H₂O₂) or final 4 h (for Antimycin A) of RF exposure, as described in Fig. 1B. BRET signals were acquired immediately after RF-EMF exposure, allowing real-time monitoring of mitochondrial ROS levels. (**G**–**I**) Box plots summarize the quantitative effects of RF-EMF exposure on basal BRET signal (**G**), pEC50 values of H_2_O_2_ and Kp372-1 (**H**), and maximal efficacy for each ROS inducer (**I**) across the different SAR conditions. Due to its limited potency, Antimycin A efficacy was calculated based only on responses to the two highest concentrations (100 and 300 µM). Statistical analysis was performed using the Kruskal–Wallis and Mann–Whitney tests. No significant differences were observed between RF-exposed and sham groups (n = 8–9 depending on condition). n.s.: not significant.

In this study, two variants of ROBINy were employed: one targeted to the mitochondrial matrix via a specific localization sequence and the other localized ubiquitously in the cytoplasm^30^. These probes were transiently expressed in XP6BE fibroblasts before exposing the cells to 5G-modulated 3.5 GHz RF-EMF signals at SAR levels of 0.08 W/kg and 4 W/kg for 24 h, or sham-exposed. At the indicated time before the end of the exposure, the cells were challenged with increasing concentrations of three different ROS inducers (Fig. 1B): KP372-1, an Akt inhibitor that targeted the redox enzyme, NAD(P)H:quinone oxidoreductase 1 (NQO1), to induce extensive ROS generation^36^; H₂O₂, a stable ROS that diffuses across membranes and generates highly reactive hydroxyl radicals^37^; and Antimycin A, a mitochondrial complex III inhibitor that promotes superoxide production by disrupting electron transport^38^. This approach aimed to investigate potential synergistic effects between RF-EMF and various ROS production pathways. BRET readings were then acquired at the end of RF exposure to assess the cytoplasmic (Fig. 2) and mitochondrial (Fig. 3) redox state in live cells.

As expected, the addition of each ROS inducer at increasing concentrations to ROBINy-transfected cells led to a dose-dependent increase in the BRET signal, regardless of whether the probe was localized in the cytoplasm (Fig. 2A–F) or mitochondria (Fig. 3A–F), reflecting the anticipated rise in ROS production in both compartments. However, the apparent potency of the three compounds remained low, with half-maximal effective concentrations (EC₅₀) in the tens of micromolar range for KP372-1 and in the millimolar range for H₂O₂ (Figs. 2H and 3H for the cytoplasmic and mitochondrial probes, respectively). For Antimycin A, no saturation of the BRET signal was observed within the tested concentration range, precluding accurate determination of EC₅₀ or maximal efficacy. Therefore, the efficacy of Antimycin A in inducing cytoplasmic and mitochondrial ROS production was quantified at fixed concentrations of 100 µM and 300 µM, respectively (Figs. 2I and 3I).

Although the dose–response curves for KP372-1, H₂O₂, and Antimycin A appeared identical between sham and RF-exposed cells, whether using the cytoplasmic or mitochondrial ROBINy probes (Figs. 2A–F and 3A–F), we performed a detailed statistical analysis to formally confirm the absence of any exposure-related effect. As shown in Figs. 2G–I and 3G–I, statistical analysis confirmed that RF-EMF exposure neither significantly altered basal cytoplasmic or mitochondrial BRET signals, nor affected the potency or maximal efficacy of KP372-1, H₂O₂, or Antimycin A in inducing ROS production. These data collectively demonstrate that (i) exposure to 5G signals alone at the tested SAR levels does not perturb steady-state cellular redox homeostasis or activate ROS-mediated signaling pathways, and (ii) no synergistic interaction exists between 5G-modulated 3.5 GHz RF-EMF and chemical ROS inducers in modulating oxidative stress responses in XP6BE fibroblast cells.

### Evaluating the potential of 5G RF-EMF to induce adaptive responses to oxidative stress in XP6BE fibroblasts

In order to next assess whether 5G-modulated 3.5 GHz RF-EMF could trigger an AR in XP6BE fibroblasts, we sought a suitable positive control capable of reliably inducing such a response. Given its dual role as both a known genotoxic environmental contaminant and a clinical therapeutic agent, As₂O₃ was selected. Arsenic compounds are widely recognized environmental toxicants due to contamination in drinking water, as well as potent carcinogens^39^. Notably, As₂O₃ is clinically utilized in the treatment of specific cancers, such as acute promyelocytic leukemia^40^. Moreover, previous studies have demonstrated that exposure to low, sub-toxic concentrations of As₂O₃ effectively induces an AR by upregulating multiple antioxidant and redox-related genes in skin cells^41^. Thus, we hypothesized that As₂O₃ could serve both as a positive control to induce an AR (when used as AD) and as CD to confirm the occurence of such an AR following RF-EMF exposure.

**Fig. 4:**
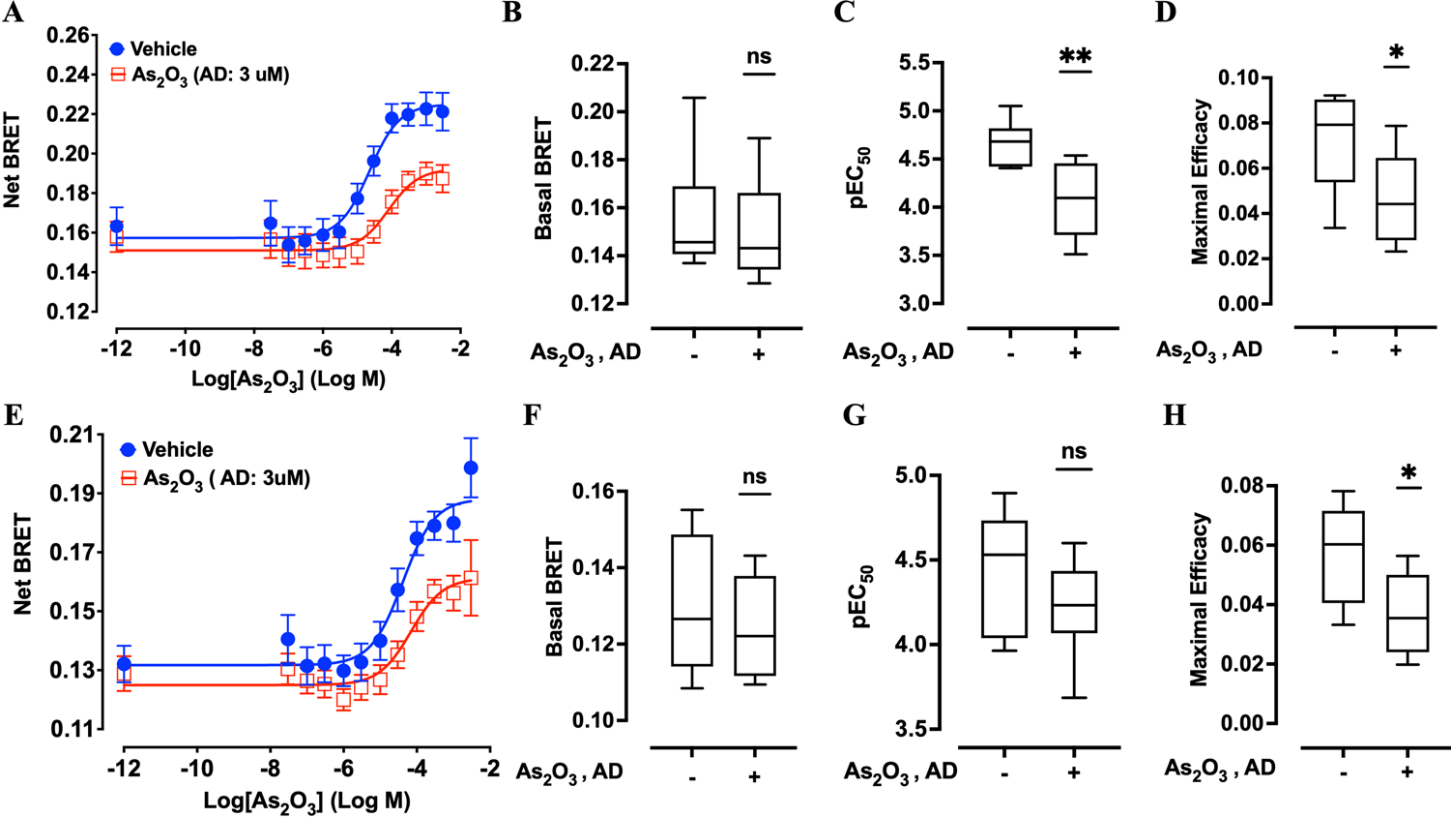
Adaptive response to arsenic trioxide (As₂O₃) in XP6BE human fibroblasts expressing cytoplasmic or mitochondrial ROBINy BRET probes. Cells were pretreated with 3 µM As₂O₃ for 20 h to induce an adaptive dose (AD), followed by a 30-min challenging dose (CD) with increasing concentrations of As₂O₃, as shown in Fig. 1C. Panels A–D show the results obtained with the cytoplasmic BRET probe: dose–response curves (**A**), basal BRET signal (**B**), pEC₅₀ values of As_2_O_3_ (**C**), and maximal efficacy of As_2_O_3_ (**D**). Panels E–H show the corresponding data for the mitochondrial probe: dose–response curves (**E**), basal BRET signal (**F**), pEC₅₀ values of As_2_O_3_ (**G**), and maximal efficacy of As_2_O_3_ (**H**). A significant decrease in pEC₅₀ (panel C) and maximal efficacy (panels D and H) was observed in cells pretreated with the adaptive dose, demonstrating a measurable adaptive response to As₂O₃. Statistical analysis was performed using the Mann–Whitney test (*p < 0.05, **p < 0.01; n = 8–9 depending on experimental condition). n.s.: not significant.

Using real-time cellular impedancemetry, we first determined that 3 µM represents the highest non-toxic concentration of As₂O₃ for XP6BE fibroblasts, as higher doses induced a rapid decline in the cell index upon addition (Supplementary Fig. 1). Based on this threshold, XP6BE fibroblasts transiently expressing either cytoplasmic or mitochondrial ROBINy BRET-based ROS sensors were exposed to 3 µM As₂O₃ for 20 h, serving as AD, or were mock-treated. Increasing concentrations of As₂O₃ were subsequently administered for 30 min as CD (Fig. 1C). BRET measurements were then acquired to quantify ROS production in both cytoplasmic and mitochondrial compartments. The results demonstrated that challenging the cells with As₂O₃ dose-dependently increased the BRET signal in mock-treated cells, reaching a plateau with pEC₅₀ values of 4.64 ± 0.18 in the cytoplasm (Fig. 4A) and 4.38 ± 0.18 in the mitochondria (Fig. 4E). Pre-treatment with the AD of As₂O₃ did not alter basal BRET signals (Fig. 4B and F) but shifted the dose–response curve rightward by approximately half a log, as reflected by reduced pEC₅₀ values in both cytoplasm and mitochondria (Fig. 4C and G). Moreover, after pre-treatment with the AD of As₂O₃, the maximal efficacy of As₂O₃ in triggering ROS production was notably diminished in both compartments (Fig. 4D and H), underscoring the protective effect of AR.

**Fig. 5:**
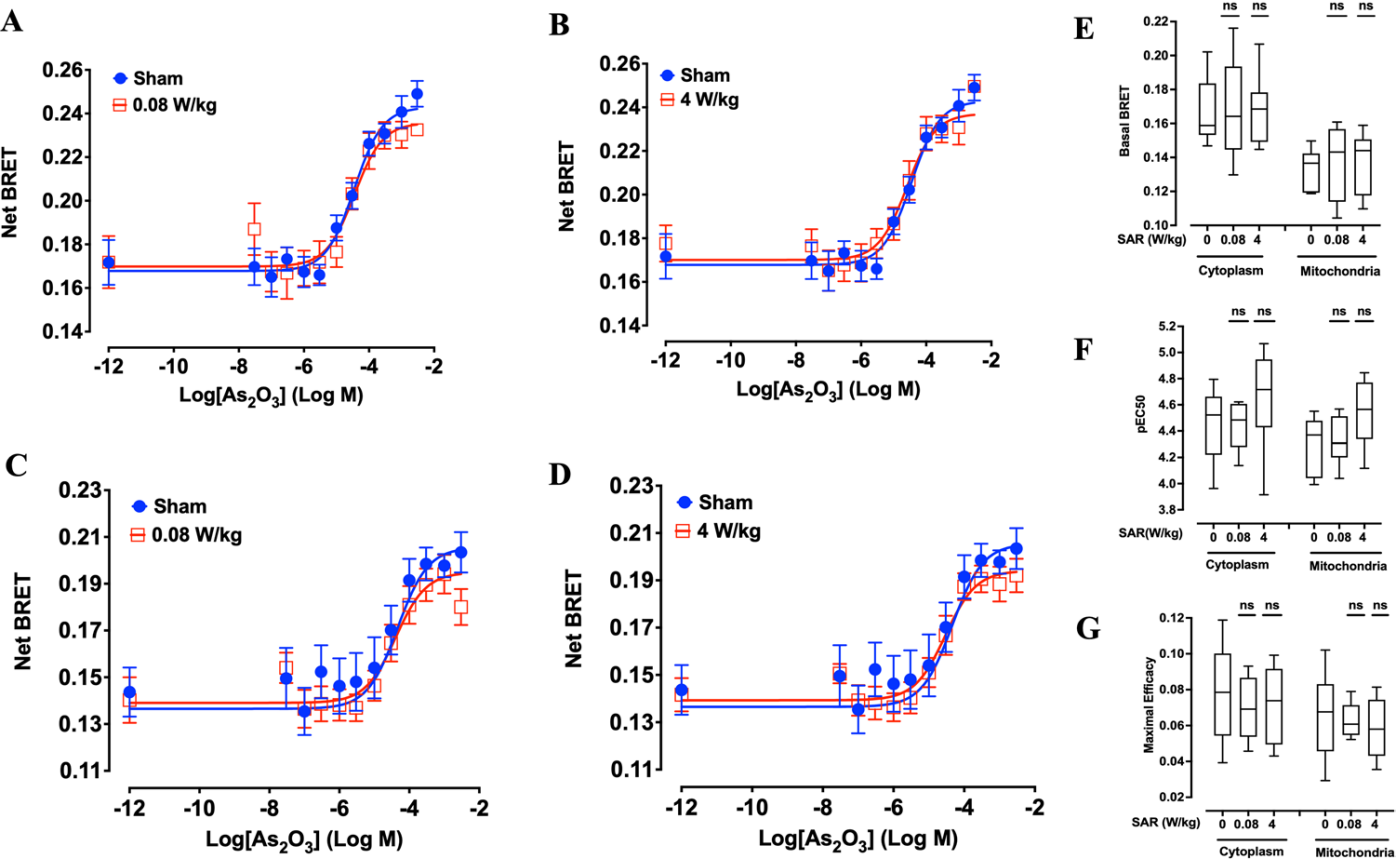
Assessment of potential RF-EMF-induced adaptive response in XP6BE fibroblasts. Cells were exposed to sham or 5G-modulated 3.5 GHz RF-EMF at 0.08 or 4 W/kg for 20 h, followed by a 3 h resting phase. Then, increasing challenging dose (CD) concentrations of As₂O₃ were applied for 30 min before BRET measurement, as illustrated in Fig. 1D. (**A** and **B**) Dose response curves of As_2_O_3_ obtained with XP6BE fibroblast cells expressing the cytoplasmic ROBINy and being exposed to RF-EMF at SAR of 0.08 W/kg (**A**) or 4W/kg (**B**). (**C** and **D**) Dose response curves of As_2_O_3_ obtained with XP6BE fibroblast cells expressing the mitochondrial ROBINy BRET probe and being exposed to RF EMF at SAR of 0.08 W/kg (**C**) or 4 W/kg (**D**). (**E**–**G**) Box plots showing basal BRET (**E**), pEC50 of As_2_O_3_ (**F**), and maximal efficacy of As_2_O_3_ (**G**) across the different SAR conditions. No adaptive response was observed following RF-EMF exposure, in contrast to As₂O₃-induced adaptation (Fig. 4). Statistical analysis was conducted using the Kruskal–Wallis Test. n = 8–9 depending on experimental conditions. *n.s*.: not significant.

To evaluate whether exposure to 5G-modulated 3.5 GHz RF-EMF could elicit an AR in XP6BE fibroblasts, cells transiently transfected with either cytoplasmic or mitochondrial ROBINy BRET-based ROS sensors were first sham-exposed or exposed to RF-EMF at SAR of 0.08 or 4 W/kg for 20 h, followed by a 3-h recovery period without exposure^17^ (Fig. 1D). Subsequently, cells were challenged with increasing concentrations of As₂O₃ to assess potential alterations in ROS production (Fig. 5). As shown in Fig. 5A–D, the dose–response curves of As₂O₃-induced ROS production in both cytoplasmic and mitochondrial compartments remained similar between sham and RF-EMF exposed cells. Detailed statistical analysis confirmed that neither the basal BRET signals (Fig. 5E), nor the pEC₅₀ values (Fig. 5F), nor the maximal efficacy (Fig. 5G) of As₂O₃-induced ROS production, whether in the cytoplasm or mitochondria, were significantly different between sham and 5G RF-EMF exposure at either SAR level tested. Collectively, these results indicate that pre-exposure to 5G-modulated 3.5 GHz RF-EMF does not trigger an AR capable of modulating oxidative stress induced by As₂O₃ in XP6BE fibroblasts.

### Effects of 5G‐modulated 3.5 GHz RF‐EMF exposure and UV-B irradiation on DNA repair machinery in HaCaT keratinocytes

We finally investigated whether exposure to 5G-modulated 3.5 GHz RF-EMF could nonetheless contribute to genomic alterations by modulating cellular responses to a well-characterized genotoxic agent. To address this question, we adopted an original approach by specifically evaluating whether RF-EMF exposure could interfere with the DNA repair machinery itself, thereby altering the resolution of DNA lesions induced by UV-B irradiation.

Keratinocytes were selected as the cellular model due to their location in the outermost layer of the skin and their critical role as the first line of defense against environmental stressors, including UV radiation and RF-EMF. Unlike dermal fibroblasts, keratinocytes are directly and chronically exposed to UV-B, and their DNA damage response is critical. UV-B radiation (280–315 nm) is one of the most potent environmental DNA-damaging agents, capable of inducing DNA lesions such as CPDs and pyrimidine (6–4) pyrimidone photoproducts (6-4PPs). Unrepaired photoproducts disrupt essential cellular processes by blocking replication and transcription, causing cytototoxic and genotoxic effects, and ultimately leading to inflammation, hyperplasia, premature skin aging, and potentially carcinogenesis^42^. For this reason, we focused our investigation on CPDs, which are predominantly repaired via the nucleotide excision repair (NER) pathway^43^.

**Fig. 6:**
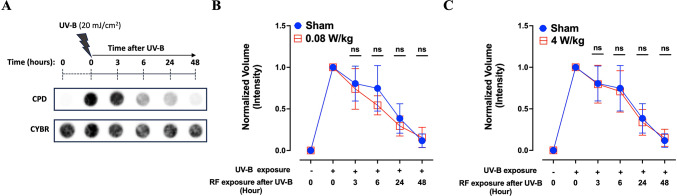
Evaluation of DNA repair following UV-B and RF-EMF exposure in HaCaT human keratinocytes. (**A**) Dot-blot analysis of CPD (Cyclobutane Pyrimidine Dimers) at various time points (0–48 h) post UV-B exposure (20 mJ/cm^2^). (**B**, **C**) Quantification of CPD repair kinetics following UV-B exposure and subsequent RF-EMF exposure at 0.08 W/kg (**B**) or 4 W/kg (**C**). RF-EMF exposure did not significantly affect DNA repair kinetics. Statistical analysis was conducted using the Mann–Whitney Test between sham and RF-EMF exposed cells (n = 7). *n.s*.: not significant.

To establish appropriate experimental conditions, HaCaT keratinocytes were first exposed to a range of UV-B doses (10, 20, 40, 70, and 100 mJ/cm^2^), and both the initial levels of UVB-induced CPD formation and their subsequent repair kinetics were evaluated. All tested doses resulted in detectable CPD formation, with varying levels of damage (data not shown). A dose of 20 mJ/cm^2^ was selected for subsequent experiments as it reliably induced CPD lesions that were efficiently repaired in the next 48 h (Fig. 6A), while maintaining approximately 75% cell viability (Supplementary Fig. 2).

We then assessed whether 5G-modulated 3.5 GHz RF-EMF exposure affected the ability of HaCaT cells to repair UV-B-induced DNA damage. Cells were first irradiated with UV-B (20 mJ/cm^2^), followed by sham exposure or RF-EMF exposure at 0.08 or 4 W/kg for up to 48 h. Samples were collected prior to UV-B irradiation, immediately after UV-B irradiation, and at multiple time points during sham or RF exposure. CPD levels were revealed using an immuno-dot blot assay and quantified (Fig. 6A). As shown in Fig. 6B and C, CPD lesions were progressively repaired over time under all experimental conditions, with near-complete resolution observed at 48 h. Importantly, no significant differences were detected in the kinetics of CPD repair between sham-exposed and RF-EMF-exposed cells at either SAR level (Fig. 6B and C). These results indicate that 5G-modulated 3.5 GHz RF-EMF does not interfere with the repair efficiency of UV-B-induced CPDs in HaCaT keratinocytes.

## Discussion

This study evaluated the potential non-thermal biological effects of 5G-modulated 3.5 GHz RF-EMF exposure on oxidative stress and DNA repair mechanisms in human skin cells.

We employed the BRET-based ROS probe ROBINy (Fig. 1A) to detect real-time ROS production simultaneously in cytoplasmic and mitochondrial compartments of human fibroblasts exposed to 5G-modulated RF-EMF at SAR levels of 0.08 and 4 W/kg. Neither basal ROS levels, nor the potency or maximal efficacy of three classical ROS inducers (H₂O₂, KP372-1, and Antimycin A), was affected by RF exposure (Fig. 2, Fig. 3). Importantly, these ROS inducers were specifically chosen because they target different cellular mechanisms, including general oxidative stress (H₂O₂), inhibition of NAD(P)H:quinone oxidoreductase (KP372-1), and mitochondrial electron transport chain disruption (Antimycin A), thus covering a broad range of oxidative pathways. Additionally, by assessing responses simultaneously in two distinct cellular compartments—cytoplasm and mitochondria—we robustly evaluated the potential of RF-EMF to modulate ROS signaling at multiple biological levels. These comprehensive data strongly confirm that 5G-modulated RF-EMF does not alter oxidative stress signaling, even in the presence of diverse exogenous oxidative challenges. The real-time dimension of our investigation significantly advances previous studies that typically measured ROS production post-exposure. Our results strongly support previous reports showing no significant effects of RF exposure on oxidative stress markers^44–48^ and align with the recent conclusion by the SSM 2021–2025 reports^15^ and the recent systematic review by Meyer et al.^16^.

Interestingly, several recent studies have also explored potential effects of RF-EMF signals allocated to the 5G band, particularly in the 26–28 GHz range. Kim et al. (2022) investigated the biological impact of 28 GHz electromagnetic radiation—a frequency designated for 5G—and reported reduced pigmentation and ROS production in murine melanoma cells and a pigmented skin model^49^. However, the signal was not confirmed to be 5G-modulated, and neither SAR values nor thermal controls were reported. Matsumoto et al. (2025) examined whole-body exposure of rats to 28 GHz waves and observed alterations in corticosterone and noradrenaline levels, but only incident power densities were provided, with no assessment of tissue heating^50^. In contrast, Sannino et al.^51^ exposed human keratinocytes and fibroblasts to both modulated and unmodulated 26.5 GHz signals under rigorously controlled dosimetric and thermal conditions, and found no significant changes in ROS levels, cell viability, or DNA integrity. These findings underscore the importance of precise dosimetry, modulation description, and thermal control when evaluating the biological effects of high-frequency RF-EMFs, including those used in 5G. Notably, several research groups have emphasized a negative correlation between the likelihood of detecting biological effects and the methodological quality of bioelectromagnetic studies^52^.

In a second time, we assessed the potential for RF-EMF to trigger an AR. Mechanistically, studies have proposed that RF-EMF-induced AR is associated to mild oxidative stress triggering the upregulation of antioxidant enzymes such as catalase and glutathione peroxidase, as well as autophagy and DNA repair pathways^53–55^. For example, He et al.^56^ found that poly (ADP-ribose) polymerase-1 (PARP-1), a key enzyme in DNA repair, was activated following RF exposure, suggesting a protective mechanism at the molecular level. Falone et al.^17^ observed that RF-induced AR could reduce oxidative DNA damage in neuroblastoma cells when challenged with additional oxidative stressors. Although earlier studies have described AR induced by RF exposure under specific conditions^22–24,54^, we found no evidence of such an effect following pre-exposure to 3.5 GHz RF-EMF. In contrast, cells preconditioning with AD of As₂O₃ followed by increasing CD of As_2_O_3_ clearly triggered an AR, validating the sensitivity and dynamic range of our assay (Fig. 4). The absence of any comparable difference between the CD dose-responses obtained following sham or RF-EMF exposure indicates that 5G-modulated RF-EMF does not induce detectable AR in our experimental model. Although we employed a similar RF-EMF-induced AR protocol to that described by Falone et al.^17^, particularly regarding the timing of the AD and CD treatments, our study differs in several key aspects: the cell type investigated, the biological endpoints assessed, and the characteristics of the RF-EMF exposure, including frequency, modulation, and specific absorption rate (SAR) level. Moreover, the absence of RF-EMF induced AD on real-time ROS production is not necessarily in contradiction with the so-called RF-EMF adaptive response on DNA damage usually described.

In terms of genotoxicity, Romeo et al.^27^ recently conducted a systematic review of in vitro studies on RF-EMF-induced DNA damage in mammalian cells. Their analysis, which included 159 articles and over 1,100 independent experiments, found that 80% of experiments reported no significant genotoxic effects of RF-EMFs, while the remaining studies showed inconsistent results with varying methodological quality. The review concluded that, based on the available data, there is low confidence in the hypothesis that RF-EMFs induce genotoxic effects in mammalian cells, particularly irreversible DNA damage. Similarly, Kaur et al. (2023) examined RF-EMF-related genotoxic risks in male reproductive health and found that while some studies report increased DNA fragmentation and chromosomal aberrations in sperm cells, others find no significant impact^57^. This underscores the variability in experimental outcomes and the need for standardized methodologies as mentioned by several authors^27,52^. Of note, in terms of exposure to RF-EMF, we followed such quality criteria using rigorous dosimetry, proper sham controls, and long-term exposure assessment in vitro^27^.

Based on the conclusion of these recent reviews on RF-EMF induced genotoxicity, we wanted to further investigate whether RF-EMF interferes with the DNA repair machinery following UV-B irradiation, a known inducer of CPDs, in HaCaT keratinocytes. Using immuno-dot blot analysis, we observed normal kinetics of CPD repair, with no detectable differences between sham and RF-exposed cells at multiple time points (3, 6, 24, 48 h post-UV irradiation), and whatever the SAR tested (Fig. 6). These findings corroborate previous in vitro studies reporting no significant effects of RF-EMF on DNA integrity or repair.

On the other hand, our results are in contradiction with some studies such as Baohong et al.^58^, who tested four genotoxic chemicals and showed that RF-EMF (GSM-modulated 1.8 GHz, 3 W/kg, 3 h) could interact with only two of them—mitomycin C and 4-nitroquinoline-1-oxide (4NQO)—to increase DNA damage in human lymphocytes. Interestingly, 4NQO is considered a UV-mimetic agent which, like UV-B, induces bulky DNA lesions primarily repaired via the NER pathway. When lymphocytes were treated with 0.5 µM 4NQO followed by RF-EMF exposure, DNA damage was significantly elevated compared to 4NQO treatment alone, whereas other doses (0.25, 1, and 2 µM) showed no such synergistic effect. This protocol is similar to ours in terms of post-damage RF-EMF exposure and the use of NER-repaired lesions as endpoints. However, in addition to the DNA damage agent used, important differences must be acknowledged. Baohong et al. used human lymphocytes and a comet assay to detect DNA strand breaks^58^, whereas our study employed HaCaT keratinocytes and a dot blot assay to detect CPD lesions. Moreover, the RF-EMF exposure systems differed in frequency (1.8 vs. 3.5 GHz), modulation type (GSM vs. 5G), and SAR values (3 W/kg for 3 h vs. 0.08 or 4 W/kg for up to 48 h), all of which could influence the biological outcomes. These discrepancies highlight the need for standardized experimental protocols and suggest that potential RF-EMF interactions with genotoxic agents may be cell type-, dose-, and endpoint-specific. Thus, although some studies reported potential synergistic effects between RF-EMF and genotoxic agents such findings are inconsistent and may be dependent on both chemicals and cell types.

As a result, research on the effects of RF-EMF exposure on oxidative stress and DNA damage or repair continues to be largely contradictory. The field remains constrained by the absence of a unifying theoretical model that could bridge experimental observations with established principles of molecular and cellular biology.

In summary, our investigation—combining novel biosensing technology with robust experimental design—demonstrates that 5G-modulated 3.5 GHz RF-EMF does not affect oxidative stress levels, trigger adaptive response, or interfere with DNA repair processes in human skin cells at steady temperature. These results are consistent with current assessments by international experts and provide meaningful data for an evidence-based risk assessment of RF-EMF exposure in the context of 5G deployment. Nevertheless, it is important to note that our findings are based on acute in vitro exposure protocols lasting up to 24–48 h. While such durations are commonly used to assess direct cellular responses, they do not fully recapitulate the chronic or long-term exposures experienced in real-life settings. Therefore, future studies will be necessary to confirm these findings in more physiologically relevant models, including long-term or repeated exposures in 3D reconstructed skin or in vivo systems.

## Supplementary Information

Supplementary Information 1.

Supplementary Information 2.

Supplementary Information 3.

## Data Availability

The datasets used and/or analyzed during the current study are available from the corresponding author on reasonable request.
