# Supplementary material for: Impact of in vitro exposure to 5G-modulated 3.5 GHz fields on oxidative stress and DNA repair in skin cells
Source: Sci Rep. 2025 Aug 25;15:31214. doi: 10.1038/s41598-025-15090-w (PMC12379245; doi:10.1038/s41598-025-15090-w)
Supplement: Supplementary file 1 — Supplementary Information 1. [file 41598_2025_15090_MOESM1_ESM.docx]

**Supplementary Figures Legends**

**Supplementary figure 1:** Real-time impedance analysis of XP6BE fibroblasts exposed to increasing concentrations of arsenic trioxide (As₂O₃). Cell Index (CI) was monitored continuously over 72 hours using the xCELLigence RTCA platform. As₂O₃ was added at 52 hours post-seeding (indicated by the vertical dashed line) at final concentrations ranging from 1 µM to 1 mM, with H₂O as control (Vehicle). Almost no effect on cell viability is observed up to 3µM of As_2_O_3_. A biphasic response was observed at intermediate concentrations (10–30 µM), characterized by an initial increase in CI (primary response), followed by a delayed concentration-dependent CI decrease indicative of cytotoxicity. At higher concentrations (≥100 µM), rapid and irreversible loss of CI was observed, reflecting acute cytotoxic effects. The figure shows one representative experiment out of 3 performed in the same conditions.

**Supplementary figure 2: (A)** Viability test by Trypan Blue was perfomed on HaCaT cells at different time points after UV-B irradiation. **(B)** Cyclopyrimidine dimer (CPD) levels were quantified in the HaCaT cell line by immuno-dot blot analysis at different time points after UV-B irradiation.
