# Supplementary figures and images for: Impact of in vitro exposure to 5G-modulated 3.5 GHz fields on oxidative stress and DNA repair in skin cells
Source: Sci Rep. 2025 Aug 25;15:31214. doi: 10.1038/s41598-025-15090-w (PMC12379245; doi:10.1038/s41598-025-15090-w)

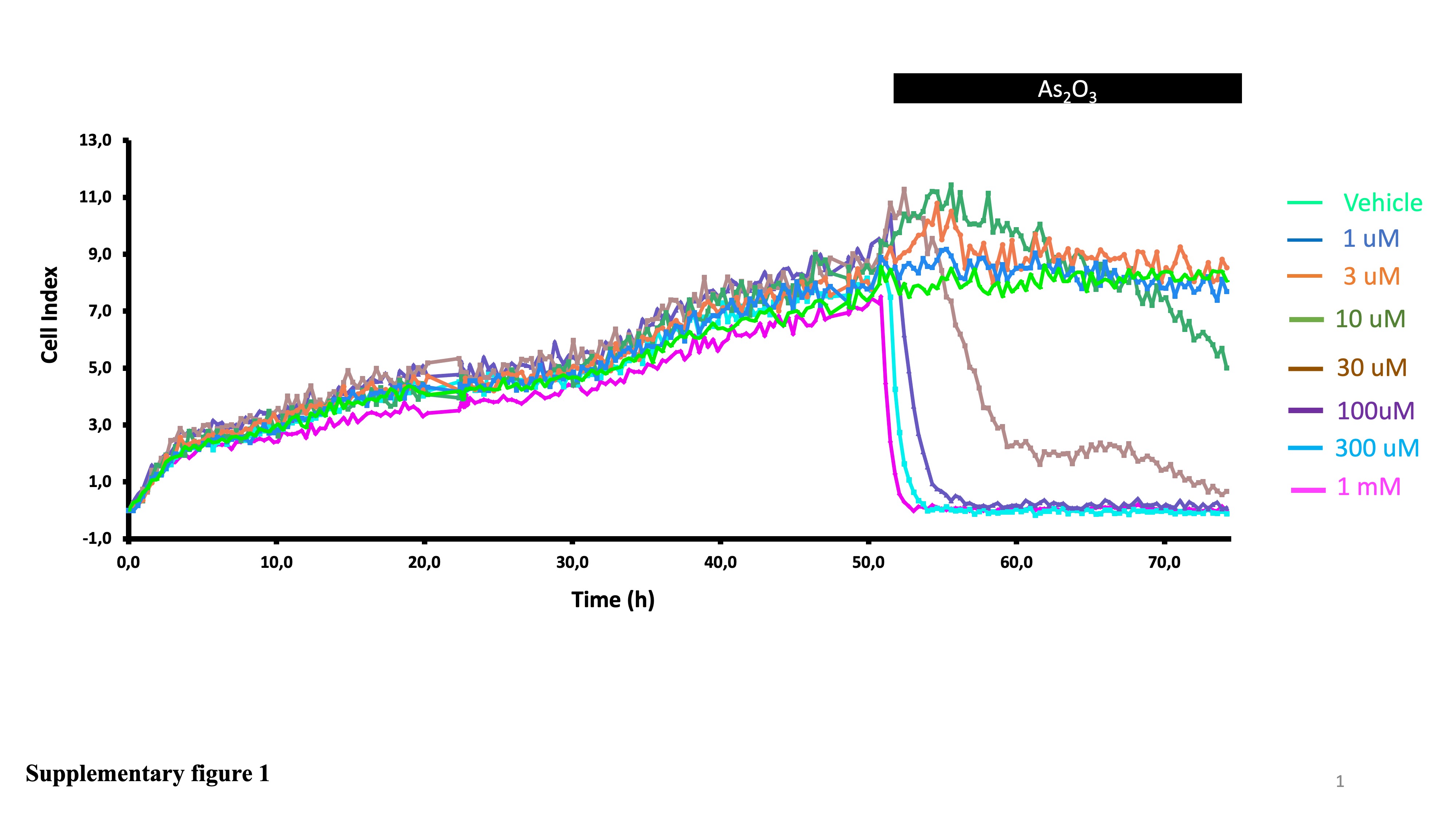

Supplement: Supplementary file 2 — Supplementary Information 2. [file 41598_2025_15090_MOESM2_ESM.jpg]

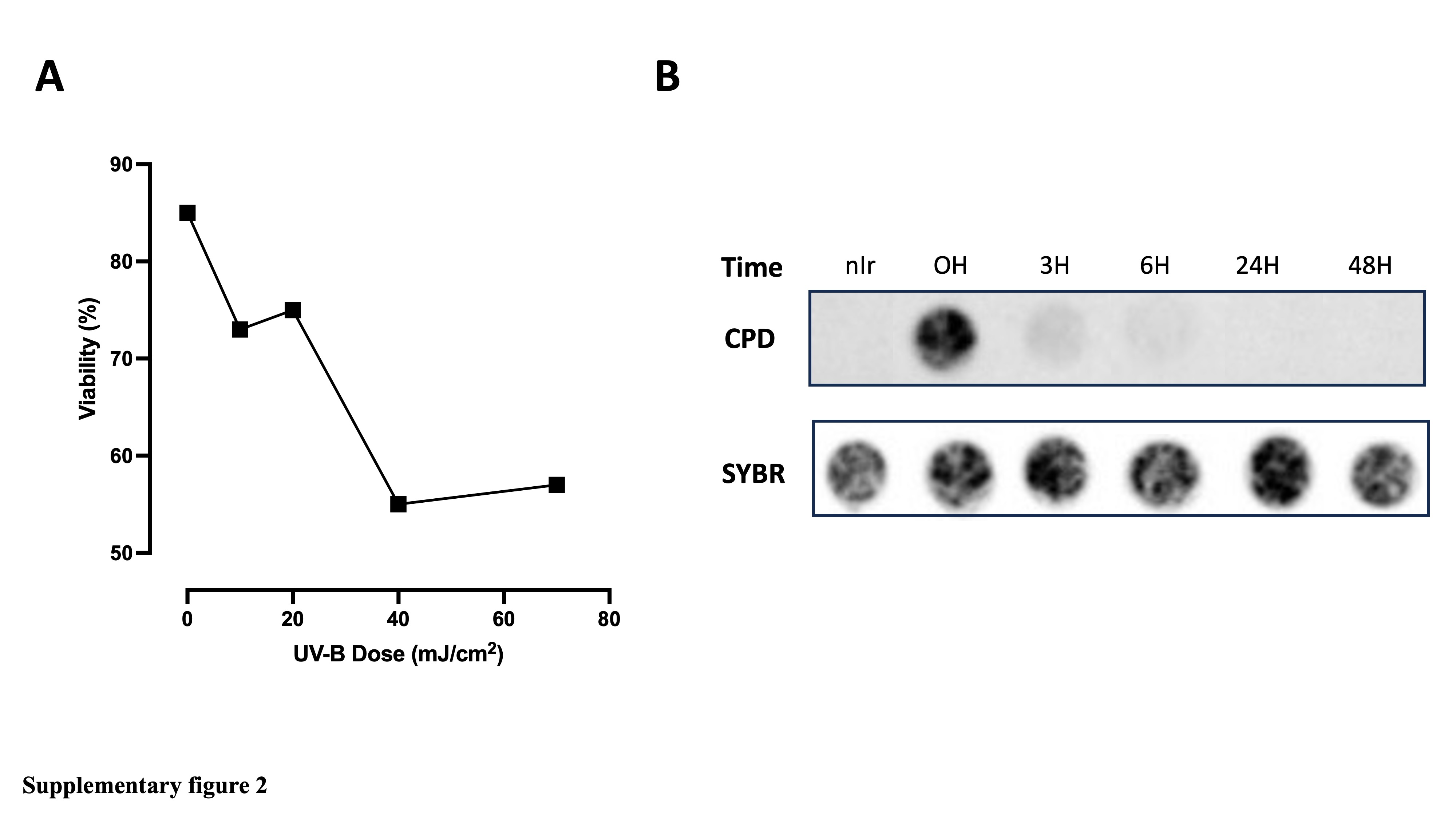

Supplement: Supplementary file 3 — Supplementary Information 3. [file 41598_2025_15090_MOESM3_ESM.jpg]
